# Biofilms from *Klebsiella pneumoniae*: Matrix Polysaccharide Structure and Interactions with Antimicrobial Peptides

**DOI:** 10.3390/microorganisms4030026

**Published:** 2016-08-10

**Authors:** Monica Benincasa, Cristina Lagatolla, Lucilla Dolzani, Annalisa Milan, Sabrina Pacor, Gianfranco Liut, Alessandro Tossi, Paola Cescutti, Roberto Rizzo

**Affiliations:** Department of Life Sciences, University of Trieste, Via L. Giorgieri 1, Trieste I-34127, Italy; mbenincasa@units.it (M.B.); clagatolla@units.it (C.L.); ldolzani@units.it (L.D.); annalisa.milan@phd.units.it (A.M.); pacorsab@units.it (S.P.); gianliut@gmail.com (G.L.); atossi@units.it (A.T.); pcescutti@units.it (P.C.)

**Keywords:** *Klebsiella pneumoniae*, biofilm matrix, matrix polysaccharides, antimicrobial peptides

## Abstract

Biofilm matrices of two *Klebsiella pneumoniae* clinical isolates, KpTs101 and KpTs113, were investigated for their polysaccharide composition and protective effects against antimicrobial peptides. Both strains were good biofilm producers, with KpTs113 forming flocs with very low adhesive properties to supports. Matrix exopolysaccharides were isolated and their monosaccharide composition and glycosidic linkage types were defined. KpTs101 polysaccharide is neutral and composed only of galactose, in both pyranose and furanose ring configurations. Conversely, KpTs113 polysaccharide is anionic due to glucuronic acid units, and also contains glucose and mannose residues. The susceptibility of the two strains to two bovine cathelicidin antimicrobial peptides, BMAP-27 and Bac7(1–35), was assessed using both planktonic cultures and biofilms. Biofilm matrices exerted a relevant protection against both antimicrobials, which act with quite different mechanisms. Similar protection was also detected when antimicrobial peptides were tested against planktonic bacteria in the presence of the polysaccharides extracted from KpTs101 and KpTs113 biofilms, suggesting sequestering adduct formation with antimicrobials. Circular dichroism experiments on BMAP-27 in the presence of increasing amounts of either polysaccharide confirmed their ability to interact with the peptide and induce an α-helical conformation.

## 1. Introduction

An increasing number of pathogens are acquiring multi drug resistance (MDR) to different classes of antibiotics, a particularly relevant issue for infections acquired in hospital settings. The European Centre for Disease Prevention and Control reported that in 2009, MDR infections caused 25,000 deaths in Europe and estimated an economic burden, resulting from healthcare costs and productivity losses, of at least €1.5 billion [1]. Today, due to the increase of antimicrobial resistance, these numbers are likely to be considerable higher. Bacterial species under observation include several Gram-negative ones, like *Pseudomonas aeruginosa*, *Escherichia coli*, *Klebsiella pneumoniae,* and *Acinetobacter baumannii*.

The current antibiotics arsenal comprises about 150 molecules from 17 different classes, but each of these has seen resistance development in bacteria by several possible mechanisms including: inactivation of the molecule itself, its expulsion, alteration of its target site, or reduced accessibility to the target. The latter mechanism is exacerbated by biofilm formation: biofilm matrices exert a protective role towards embedded microorganisms [2], obliging an increase in antimicrobial doses.

Antimicrobial (host defence) peptides (AMPs), widespread effectors of the innate immune response in animals, are promising leads to develop novel anti-infective agents with alternative mechanisms to drugs in clinical use. In addition to direct killing of pathogens, they can also help recruit immune cells to the site of infection and neutralize the pro-inflammatory effects of endotoxins. They have multimodal killing mechanisms, involving interaction with bacterial components different to those targeted by conventional antibiotics [3]. This multimodal action by a single molecule makes acquisition of resistance more difficult, requiring concurrent multiple alterations in different targets. AMPs have a recognized potential for generating new agents effective against MDR and/or biofilm producing pathogens, and may be particularly useful at preventing colonization of abiotic surfaces in medical devices, such as ventilators or catheters, which are potential sources of infection.

This paper describes a study on *K. pneumoniae* focused on the definition of its biofilm forming potential as well as on the structural characterization of the polysaccharides present in the biofilm matrix and on the evaluation of the activity of selected AMPs in the presence of biofilms. *K. pneumoniae* is well known to produce 77 different capsular polysaccharides (K-antigens) and 8 basic types of *O*-antigens. K-antigens are recognized virulence factors and exhibit protecting effects by contributing to the evasion of innate immunity mechanisms, such as the action of AMPs [4,5,6], which are usually investigated mainly with a focus on planktonic bacteria [7]. In addition, the anti-biofilm activity of AMPs or their derivatives has been described [8]. Despite the numerous studies on structure and function of K-antigens, very little information is reported about the *K. pneumoniae* polysaccharides constituting the biofilm matrix.

In the present study, clinical isolates of *K. pneumoniae* were collected and their cultures, obtained both in planktonic and biofilm mode, were investigated in the presence of BMAP-27 and Bac7(1–35), bovine AMPs belonging to the cathelicidin family of host defence peptides. The former is a membranolytic peptide with helical active form [9], while the latter is the 1–35 fragment of the Pro-rich extended bactenecin Bac7, which can penetrate into susceptible Gram-negative bacteria and inactivate cytoplasmic targets [10]. The selection of these two AMPs was dictated by their different mechanism of action. To deeper investigate the effect of the matrix on AMP’s action, two isolates with diverse characteristics were chosen: abundant matrix production within biofilm (BF) or flocs, and the presence of polysaccharides exhibiting very different chemistry.

## 2. Materials and Methods

### 2.1. Bacterial Strains and Culture Conditions

Two strains of *K. pneumoniae* were chosen among 30 not-correlated clinical isolates (data not shown) for their ability to form either a biofilm (BF) strongly attached to polystyrene (namely strain KpTs101) or a “floating” biofilm (namely strain KpTs113). All strains were stored at −80 °C in Luria Broth (LB) medium containing 9% DMSO. Biofilm production and antimicrobial activity assays were carried out by incubating bacteria in Müller–Hinton Broth (MHB) at 37 °C.

### 2.2. Antimicrobial Peptides

BMAP-27, Bac7(1–35) and its BODIPY fluorescently labelled derivative (Bac7(1–35)-BY) were prepared by solid phase peptide synthesis, and purified, as previously described [11,12]. BMAP-27 is a C-terminally amidated AMP with the following sequence: GRFKRFRKKFKKLFKKLSPVIPLLHL–am. Like its close analogue BMAP-28, this type of linear cathelicidin undergoes a transition from a disordered coil conformation to the biologically active α-helical one in the presence of anisotropic or membrane-like environments [13,14]. Bac7(1–35) consists of an N-terminal, 35 residue fragment of a proline rich peptide of 69 residues, and has the following sequence; RRIRPRPPRLPRPRPRPLPFPRP GPRPIPRPLPFP–OH.

### 2.3. Evaluation of the Antimicrobial Activity of Peptides against Planktonic Cells

The bacterial inoculum was incubated overnight in MHB with shaking, then diluted 1:30 in fresh MHB and incubated with shaking for approximately 2 h to the mid-log phase, before resuspending to the appropriate dose, according to the optical density (OD).

Minimum inhibitory concentration (MIC) determinations were carried out in MHB on mid-log phase bacteria (1–5 × 10^5^ CFU/mL, CFU: colony-forming units) as previously described [7]. The MIC was taken as the lowest concentration of antimicrobial peptide resulting in the complete inhibition of visible growth after 24 h of incubation.

To assess growth inhibition, bacterial growth curves were also determined using mid-log phase bacteria, starting at 1 × 10^6^ CFU/mL in MHB, in the presence of increasing peptide concentrations, monitoring the optical density at 620 nm every 10 min at 37 °C for 4 h in a microplate reader with intermittent shaking (Tecan Trading AG, Männendorf, Switzerland).

### 2.4. Biofilm Assays

Each strain was cultured overnight in MHB, diluted 1000× in the same medium and inoculated in a 96 wells polystyrene microtiter plate (200 µL/well carried out in triplicate). After 24 h of static incubation, the culture suspensions were removed and each well was gently rinsed with 200 µL of sterile saline solution. After 1 h further incubation at 60 °C to fix the BF, each well was stained with 200 µL of crystal violet 2% (CV) (Sigma, Saint Louis, MO, USA) for 15 min, rinsed with water and dried at room temperature. The amount of CV bound to BF was evaluated by measuring the OD at 570 nm after solubilisation in 200 µL of 33% acetic acid for 30 min. Results are reported as biofilm index (BI), defined as the percentage ratio between the OD_570_ after CV solubilisation and the OD_590_ of the bacterial suspension relative to the same well.

The antimicrobial activity of peptides on sessile cells was evaluated by the BF susceptibility assay [15], with minor modifications. Briefly, the BF grown on peg lids immersed in 100 µL of bacterial suspension—prepared as described above—was incubated in fresh medium containing serial dilutions of antimicrobial peptides; after 24 h, it was rinsed three times with sterile saline, transferred in fresh medium by centrifugation at 850× *g* for 20 min and further incubated for 6 h. The biofilm inhibitory concentration (BIC) was established as the lowest concentration that inhibited growth of detached cells (OD_590_ < 0.05).

### 2.5. Confocal Microscopy (CLSM) Experiments

Biofilms were grown in 12-well chambers (ibidi GmbH, Planegg, Germany) for 24 h, gently rinsed once with water and stained either with 0.1% *w*/*v* acridine orange after fixation by incubation at 60 °C for 1 h or without fixation with the LIVE/DEAD *Bac*Light Bacterial Viability Kit (Molecular Probes, Eugene, OR, USA), according to the manufacturer’s instructions. After removing the silicone gasket, coverslips were mounted and slides were examined by CLSM using a Nikon C1-SI confocal microscope. The image stacks collected by CSLM were analysed with the EZ-C1 Free Viewer (Nikon Corporation, Tokyo, Japan) and the Image J 1.47 (Wayne Resband, National Institutes of Health, Bethesda, MD, USA) softwares. The biofilm architecture was analysed using the COMSTAT software package [16]. Results are the mean of three independent experiments ± standard deviation.

### 2.6. Flow Cytometric Analysis

Flow cytometric assays were performed with a Cytomics FC 500 instrument (Beckman-Coulter, Inc., Fullerton, CA, USA) equipped with an argon laser (488 nm, 5 mW) and photomultiplier tube fluorescence detectors. Filtered light wavelength was set at 610 nm for Propidium Iodide (PI) (Sigma, Saint Louis, MO, USA) detection and at 525 nm for BODIPY (BY) detection. All detectors were set on logarithmic amplification. Optical and electronic noise were eliminated by setting an electronic gating threshold on the forward scattering detector, while the flow rate was kept at a data rate below 300 events/second to avoid cell coincidence. For each sample, at least 10,000 events were acquired and stored as list mode files.

For the uptake evaluation, cultures of mid-log phase bacteria, diluted to 1 × 10^6^ CFU/mL in MHB, were incubated at 37 °C for 10 min with 0.25 µM Bac7(1–35)-BY; after incubation, treated bacterial cells were washed three times in buffered high salt solution (10 mM sodium phosphate, 400 mM NaCl, 10 mM MgCl_2_, pH 7.2) in order to remove the fraction of peptide bound to the cell surfaces, and immediately analysed by the flow cytometer with or without a 10 min pre-incubation at room temperature with 1 mg/mL of the quencher Trypan Blue (TB) [17].

Integrity of the bacterial cell membrane was assessed by measuring the PI uptake by flow cytometry, as previously described [18]. Briefly, mid-log phase bacterial cultures, diluted at 1 × 10^6^ CFU/mL in MHB, were incubated at 37 °C for different times with different concentrations of BMAP-27. PI was added to all samples at a final concentration of 10 μg/mL and after incubation, bacterial cells were analysed by flow cytometry.

Data analysis was performed with the FCS Express 3 software (De Novo Software, Los Angeles, CA, USA).

### 2.7. Biofilm Production and Extraction and Purification of Exopolysaccharides

*K. pneumoniae* strains Ts113 and Ts101 were grown on cellulose membranes deposited on solid agar medium [19]. Cellulose membranes (Sigma, cut-off 14,000 Da) were cut in a circle the size of the Petri dish (90 mm diameter), washed first in a boiling 5% Na_2_CO_3_ solution for 15 min and then in boiling water for 15 min, autoclaved and placed over Petri dishes, containing Müller–Hinton (MH) solid medium. The excess of water was removed before seeding the bacteria. Thirty plates were used for each strain. Two spots of 10 µL of overnight liquid cultures in MH broth were deposited on the cellulose membranes and after 2 days of incubation at 30 °C, the material from each Petri dish was scraped from the membrane and recovered in 3–5 mL of 0.9% NaCl. The suspensions were centrifuged at 48,000× *g* at 4 °C for 20 min, and filter-sterilized (Millipore membranes 0.22 μm). UV spectroscopy showed the absence of proteins and nucleic acids. The solutions containing KpTs113 and KpTs101 polysaccharides were dialyzed first against 0.1 M NaCl, then water, taken to pH = 6.8 and pH = 6.7, respectively and recovered by lyophilisation. They were named EPOL Kp113 and EPOL Kp101.

### 2.8. Exopolysaccharides Composition and Glycosidic Linkages Determination

For the determination of polysaccharides composition two methods were used: (i) for neutral saccharides detection, the polysaccharides were hydrolysed with 2 M trifluoroacetic acid (TFA) at 125 °C for 1 h, followed by derivatisation of the monosaccharides to alditol acetates [20]; (ii) for uronic acid detection, the polysaccharides were subjected to methanolysis with 2 M HCl in methanol at 85 °C for 16 h, followed by derivatisation of the methyl glycosides mixtures to trimethylsilyl derivatives (TMS) using the reagent Sylon™ HTP (Sigma) [21]. The products were analysed by Gas-Liquid Chromatography GLC on a PerkinElmer Autosystem XL (PerkinElmer, Waltham, MA, USA) gas chromatograph equipped with a flame ionisation detector, a capillary column and using He as carrier gas. Alditol acetates were separated on a SP2330 column (Supelco, 30 m) with the temperature program: 200–245 °C at 4 °C/min, while for the TMS methyl glycosides a HP-1 column (Hewlett Packard, 30 m, Agilent Technologies, Santa Clara, CA, USA) was used with the temperature program: 150–280 °C at 3 °C/min. The glycosidic linkages determination was achieved by methylation analysis. The lyophilized sample was permethylated [22], hydrolysed with 2 M TFA for 1 h at 125 °C, the products reduced to alditol with NaBH_4_ and subsequently peracetylated [20] to obtain partially methylated alditol acetates derivatives. The mixture was analysed by GLC using a SP2330 or a HP-1 capillary columns. In order to quantify each sugar derivative, peak areas were corrected by the effective carbon response factor [23]. The mixture of partially methylated alditol acetates was also analysed by GLC-MS (MS = Mass spectrometry) using an Agilent Technologies 7890A gas chromatograph coupled to an Agilent Technologies 5975 C VL mass spectrometry detector, equipped with the same capillary columns and using He as carrier gas. The temperature program used was: 150–250 °C at 4 °C/min.

### 2.9. Circular Dichroism Experiments

Circular dichroism spectra were obtained using a J-600 spectropolarimeter (JASCO Europe s.r.l., Cremella, Italy), using a 2 mm optical path quartz cuvette thermostatted at 25 °C. Measurements were carried out at constant BMAP-27 concentration (20 μM) in the presence of increasing amounts of polysaccharide: EPOL Kp113 from 0 to 400 µM, moles of pentasaccharide; EPOL Kp101 from 0 to 380 µM, moles of pentasaccharide in phosphate buffer (10 mM, pH 7.4). The concentration is given in terms of pentasaccharide units, since it is the repeating unit of EPOL Kp113 polysaccharides and used also for EPOL Kp101 which contains only galactose, to allow direct comparison.

## 3. Results

### 3.1. Strains Description

The biofilm forming ability of 30 *K. pneumoniae* clinical isolates was evaluated by the crystal violet procedure and they were divided into three classes as a function of the BF index (BI): excellent: BI ≥ 95th percentile [24], good: BI between the 95th and the 50th percentile, and scarce: BI < 50th percentile. Preliminary evaluation of the susceptibility of planktonic forms towards the bovine AMPs Bac7(1–35) and BMAP-27 was carried out using the microbroth dilution method, allowing the set-up of further analyses (Table 1). The protective effect of BF towards Bac7(1–35) and BMAP-27 was tested by the biofilm inhibitory concentration (BIC) assay that gives information about protection conferred by the whole matrix. Except for strain KpTs12, results showed that sessile cells were much less susceptible to the peptides’ action than the planktonic ones (Table 1), even in the case of KpTs113, whose BF has very poor adhesive properties.

**Figure 1:**
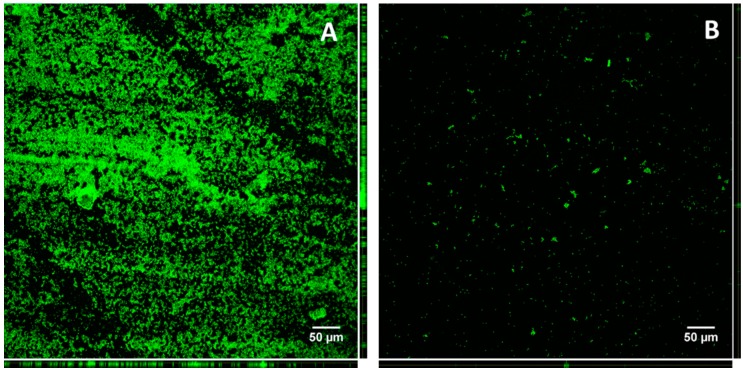
Total biofilm produced by KpTs101 (**A**); and KpTs113 (**B**) on glass slides. After 24 h growth the biofilms were fixed and stained with acridine orange 1% *w*/*v*. Z-stacks were acquired with a 20×/0.5 air objective at 1 µm intervals. Orthogonal views of Z-stacks are shown in the right and lower sides of each frame.

Two strains, both collected from urine samples, were selected for this study: one was the best biofilm producer (KpTs101) and the second one was able to produce abundant extracellular matrix, but with very low adhesion ability (KpTs113). In fact, when cultured in a liquid medium containing a glass slide, KpTs113 formed flocs [25], which detached when the slide was taken out and rinsed. Confocal microscopy, after staining with acridine orange, evidenced a large difference in the quantity of biomass adhering to the glass for the two strains (Figure 1). As a consequence, the biofilm forming ability of the KpTs113 strain was largely underestimated by the CV assay on microtiter plates, where most of the biofilm was probably lost on rinsing.

### 3.2. Growth Kinetic Assays

**Figure 2:**
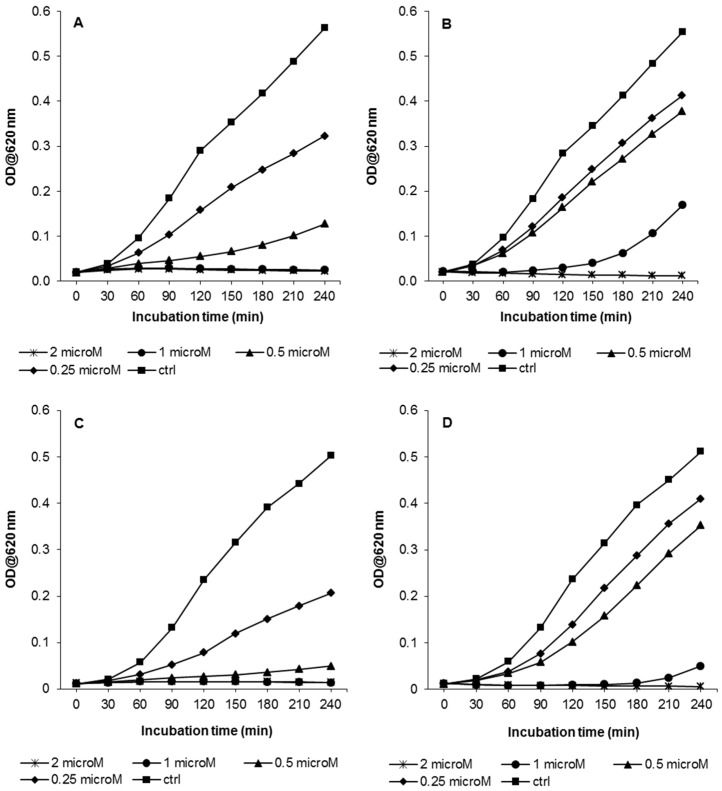
Growth kinetics of KpTs101 and KpTs113 strains in presence of Bac7(1–35) or BMAP-27. Bacterial suspensions (10^6^ cells/mL) were grown for 4 h in presence of the indicated concentrations of peptides, and the OD_620_ was measured every 10 min. The results are the means of three independent experiments. (**A**) KpTs101/Bac7(1–35); (**B**) KpTs101/BMAP-27; (**C**) KpTs113/Bac7(1–35); (**D**) KpTs113/BMAP-27.

MIC assays (Table 1) are not a very sensitive way of comparing antibacterial activity for AMPs, on different bacterial strains as they are based on serial dilutions, and furthermore, even if only just a few bacteria survive at a given peptide concentration on overnight culture, these can eventually grow and mask any difference in susceptibility. Therefore, growth kinetics assays were carried out with the two *K. pneumoniae* strains, over a shorter period of time (240 min) and in the presence of a range of sub-inhibitory to inhibitory concentrations of Bac7(1–35) and BMAP-27, by measuring the optical density of bacterial cells suspension (Figure 2). According to the similar MIC values obtained, no significant differences of susceptibility to Bac7(1–35) were evident for the two strains considered. Bac7(1–35) had inhibiting effects on bacterial growth also at sub-inhibitory concentrations (0.5–1 µM), and these were still detectable even at 0.25 µM (Figure 2A,C). Similarly, bacterial growth of KpTs101 and KpTs113 was slowed in the presence of 0.25–0.5 µM BMAP-27, significantly decreased at a peptide concentration of 1 µM, and totally inhibited at 2 µM (Figure 2B,D). From the growth kinetic data, IC_50_ (IC_50_: concentration required for 50% inhibition) values of the two peptides were calculated: Bac7(1–35) had IC_50_ of 0.2–0.3 µM and BMAP-27 of 0.6–0.7 µM, for both *Klebsiella* strains.

### 3.3. Evaluation of Bac7(1–35) Uptake in K. pneumoniae Strains

**Figure 3:**
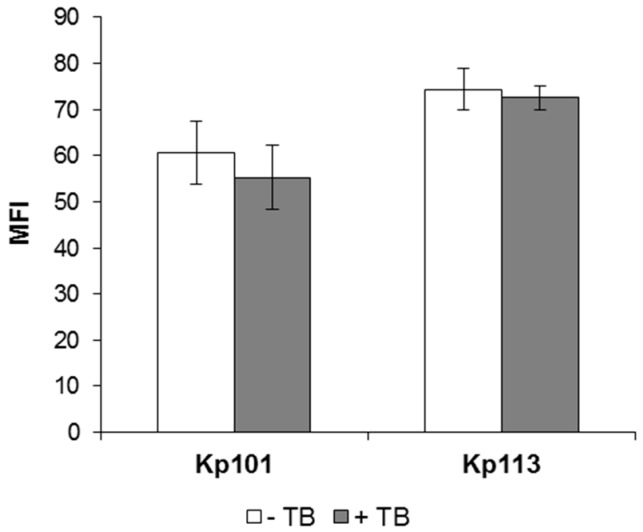
Evaluation of Bac7(1–35) internalization into *K. pneumoniae* Ts101 and Ts113 strains. Fluorescence of KpTs101 and KpTs113 cells exposed for 10 min to 0.25 µM Bac7(1–35)-BY was reported. Bacterial cells (1 × 10^6^ CFU/mL) were incubated with peptide, extensively washed, and analyzed by flow cytometry with (**grey** columns) or without (**white** columns) incubation with 1 mg/mL TB for 10 min at 37 °C. Data are expressed as the average of mean fluorescence intensity (MFI) ± SD from three independent experiments.

To investigate the correlation between the observed antimicrobial activity of Bac7(1–35) against *K. pneumoniae* with its internalization, the level of uptake of the fluorescent derivative Bac7(1–35)-BY into bacterial cell was evaluated. Peptide-treated bacteria were incubated with the quencher Trypan Blue (TB), which does not penetrate into undamaged cells and can thus quench the fluorescence only of extracellular and accessible dye-conjugated peptide. Upon treating KpTs101 and KpTs113 cells with sub-inhibitory and non-permeabilizing concentrations of Bac7(1–35)-BY for 10 min, the fluorescence intensity was not reduced by the TB quencher (<10% quenching) (Figure 3), indicating a rapid uptake of Bac7(1–35) by both strains and suggesting a substantial intracellular localization of the peptide. The mean fluorescence intensity (MFI) values for the KpTs113 strain were higher than for KpTs101, in agreement with the higher susceptibility observed for KpTs113 strain in MIC and growth kinetics assays (see Table 1 and Figure 2A,C).

### 3.4. Effect of BMAP-27 on Bacterial Membrane Integrity

**Figure 4:**
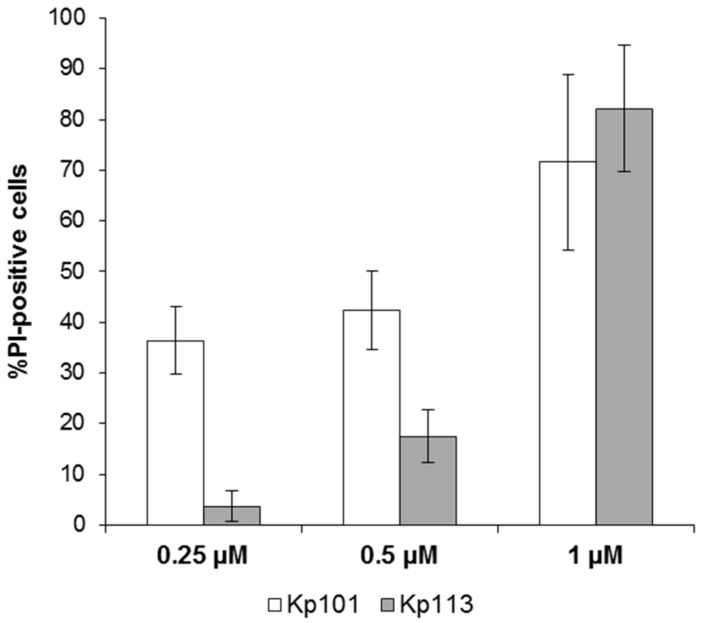
Evaluation of membrane damage induced by BMAP-27 in *K. pneumoniae* Ts101 and Ts113 strains. The percentage of damaged cells (% PI-positive cells) of KpTs101 and KpTs113 strains after 30 min incubation is reported as a function of BMAP-27 concentration. Bacterial cells (1 × 10^6^ CFU/mL) were incubated for 30 min with peptide; propidium iodide (PI) was added to each sample to final concentration of 10 µg/mL and analyzed by flow cytometry. Data are expressed as the mean ± SD for three independent experiments.

The permeabilizing effect of membrane-active BMAP-27 was evaluated in planktonic *K. pneumoniae* cells by the PI-uptake assay (Figure 4). For the KpTs101 strain, membrane damage was evident after 30 min of treatment, even at a concentration below its MIC: at 0.25 µM the percentage of damaged cells was 30%–40%, and this value rose upon increasing the peptide concentration, showing a clear dose-dependence (Figure 4, white columns). The percentages of PI-positive KpTs113 cells were significantly lower than KpTs101 after treatment with 0.25 and 0.5 µM BMAP-27, and showed a dose dependent pattern (Figure 4, grey columns). When treated with 1 µM BMAP-27, both strains reached more than 70%–80% of PI-positive cells, indicating that the activity of this peptide is principally due to its membranolytic effect (Figure 4).

### 3.5. Biofilm Formation at Sub-Inhibitory Concentrations of AMPs

As described above, *K. pneumoniae* cells exposed to sub-inhibitory concentrations of AMPs suffered to a different extent, but part of the population remained viable and was able to reach the stationary phase if incubated for 24 h. Therefore, their BF forming ability was tested under these conditions.

**Figure 5:**
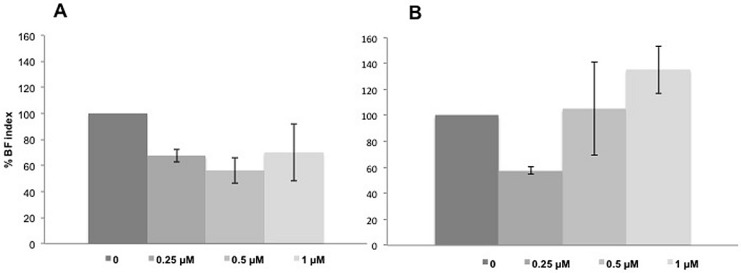
Biofilm (BF) production of KpTs101 in the presence of sub-inhibitory concentrations of Bac7(1–35) (**A**); and BMAP-27 (**B**). The BF index in the presence of AMPs after 24 h incubation was reported as percentage with respect to the control. Results are the mean of three independent experiments ± SD.

KpTs101 was grown for 24 h in microtiter wells in the presence of sub-inhibitory concentrations of Bac7(1–35) or BMAP-27 and the amount of BF produced was evaluated by CV staining. Results were expressed as BF index (Figure 5) in order to normalize the biomass produced with respect to bacterial growth. At sub-inhibitory concentrations (0.25 µM), both AMPs caused about 40% significant decrease in BF formation ones. It is noteworthy that, however, at higher concentrations, although the BF index increased, a larger variability among independent experiments was observed, probably due to the difficulty of the strains to grow at concentrations close to the MIC values, so it was not possible assess with significance if the BF was decreased (Bac7(1–35)) or even increased (BMAP-27).

### 3.6. Characterisation of the Biofilm Matrix Polysaccharide Produced by KpTs101 and KpTs113

Bacterial polysaccharides are considered the main component of biofilm matrices. On the one hand they are responsible for the setting-up of the macromolecular scaffold embedding bacterial cells. On the other hand, they may exhibit interesting biological activity due to their specific structural characteristics. For this reason, polysaccharides produced by the two *K. pneumoniae* strains were isolated from biofilms. After growing bacteria on semipermeable cellulose membranes, about 50 mg of polysaccharide produced by KpTs101 and 15 mg by KpTs113 were recovered and the samples were named EPOL Kp101 and EPOL Kp113, respectively. Their chemical characterization is hereafter briefly reported.

**EPOL Kp101**. Composition analysis as alditol acetate and trimethylsilyl methyl-glycosides (TMS) derivatives showed the presence only of galactose (Gal). The determination of the linkage position showed both 3-linked Gal*f* and 3-linked Gal*p*. These results indicated that EPOL Kp101 is a neutral polysaccharide composed of Gal in both furanose and pyranose ring structures. In addition, preliminary NMR investigation strongly indicated that it is a mixture of two d-galactan polymers (unpublished results) identical to the O-antigen polysaccharides of *K. pneumoniae* serotype O1 [26].

**Scheme 1:**
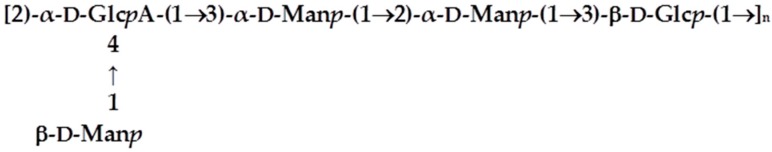
Primary structure of EPOL Kp113.

**EPOL Kp113**. Recent investigations carried out in our laboratory demonstrated that the EPOL Kp113 structure [27] is identical to the K24 capsular polysaccharide [28]. The structure is reported in Scheme 1.

Considering that the extraction procedure from biofilm of KpTs101 and KpTs113 was very mild, and not able to cleave covalent bonds, the structures defined for the polysaccharides indicated that they are both cell surface polymers released into the BF matrix.

### 3.7. Evaluation of the Protective Effect of the Matrix

In order to evaluate if the BF protective effect, reported in Table 1, could be attributed to the matrix polysaccharides, MIC assays with planktonic cells were performed in the presence of 1 mg/mL each of EPOL Kp101 and EPOL Kp113 purified from matrices. Data are reported in Table 2 (Ps column) together with MIC and BIC values from Table 1 for an easy comparison. The results do indicate that polysaccharides significantly contribute to the protective action of the matrix. To further investigate this protection mechanism, we then determined if the matrix polysaccharide interacted with the helical AMP BMAP-27 by monitoring its effect on peptide structure, using circular dichroism.

### 3.8. Circular Dichroism (CD) Spectroscopic Study

**Figure 6:**
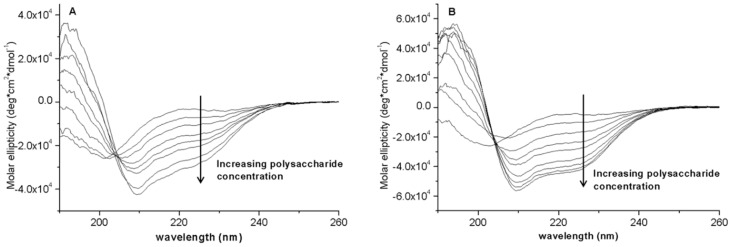
Concentration-dependent circular dichroism spectra of BMAP-27 as a function of increasing amount of (**A**) EPOL Kp113; and (**B**) EPOL Kp101. [BMAP-27] = 20 µM. Polysaccharide concentrations: EPOL Kp113 from 0 to 400 µM, moles of pentasaccharide; EPOL Kp101 from 0 to 380 µM, moles of pentasaccharide. Solvent: phosphate buffer 10 mM, pH 7.4.

The spectroscopic analysis was limited to BMAP-27 because it undergoes a transition from a random conformation to an active amphipathic α-helix in anisotropic and membrane-like environments, and this is likely correlated with its membranolytic activity [29]. Conversely, bactenecins do not show an altered conformation in the presence of membranes, or produce membrane lysis at active concentrations [19,30]. For helix-forming cathelicidins, the disordered coil conformation facilitates the approach to the membrane where they undergo the transition to an amphipathic helix conformation, allowing insertion of the hydrophobic sector into the lipid bilayer and subsequent membrane lysis via either or both a carpeting and toroidal pore mechanism [31,32]. The data collected here (Figure 6) indicates that also in the presence of both EPOL Kp101 and EPOL Kp113 the peptide underwent a conformational transition to a helical conformation. Concentration-dependent) helix induction in the presence of polysaccharide is direct evidence of the formation of a complex between the two species, and by analogy to what occurs with primate helical cathelicidins, this can lead to a sequestering effect that impedes the approach to the bacterial cytoplasmic membrane [31,32,33].

Figure 6 clearly shows the transition from a peptide disordered conformation to α-helices, whose CD spectrum is typically characterized by a double negative absorption bands centred approximately at 210–222 nm. Based on the method of Chen et al. [34] the helix content is close to 100% in the presence of EPOL Kp101 and close to 90% in the presence of EPOL Kp113. The strong helix-inducing capacity of the polysaccharides suggests that the interaction with the peptide itself is strong. Furthermore, it is not just of on electrostatic nature, as the neutral polysaccharide has the greater capacity to insure helix formation.

### 3.9. Confocal Microscopy of KpTs101 Biofilms

**Figure 7:**
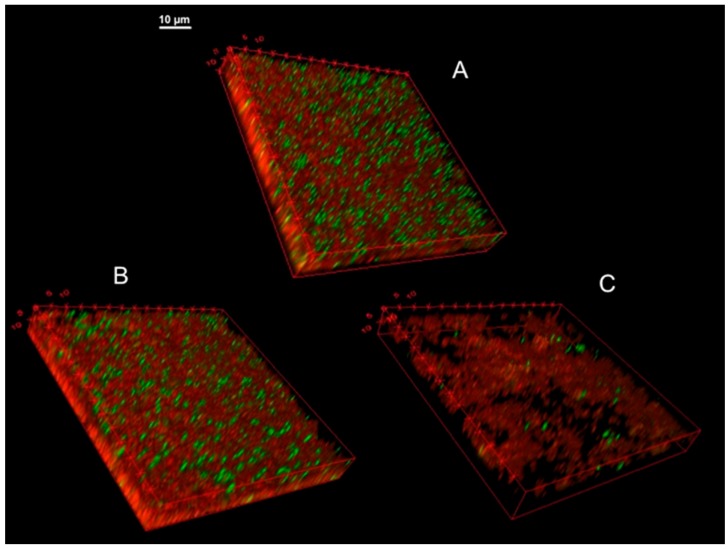
3D images of KpTs101 BF treated with AMPs. The biofilm formed after 24 h growth by KpTs101 on glass was incubated for further a 24 h in saline solution alone (**A**); or with addition of 64 µM Bac7(1–35) (**B**); or BMAP-27 (**C**), gently washed and stained with the live/dead kit: live bacteria were stained green and dead bacteria were stained red. Z-stacks were acquired with a 60×/1.4 oil objective at 0.33 µm intervals.

The effect of high concentrations of AMPs on the architecture of a mature KpTs101 biofilm was investigated with confocal microscopy by examining a 24 h grown BF treated with 64 µM AMPs. The BF was stained with a Syto9/PI mix to distinguish live from dead cells (Figure 7). In these conditions, BMAP-27 displayed an evident crumbling effect compared to the control (Figure 7C). The biofilm height was reduced by 36% ± 6%, the total biomass was reduced by 75% ± 0.2% (with 87% ± 5% decrease in total live cells), and the roughness coefficient increased 5 ± 0.2 times for live cells and up to 24 ± 1 times for dead cells, highlighting the scattered distribution of the structure.

Treatment with 64 µM Bac7(1–35) (Figure 7B) caused reduction of the biofilm height by 21% ± 14% with respect to the control (Figure 7A) while the biomass was apparently not affected. The roughness coefficient displayed a significant increase (more than 100 times), strongly suggesting that the treated structure was denser than the control. In particular, the population of dead cells was more represented in the bottom section of the biofilm. In fact, although many dead cells are usually present in the lower stacks, in the observed specimens 40% ± 4% of red staining was detected in the lowest quarter of the structure, while 29% ± 0.4% was measured in the same portion of the control sample.

## 4. Discussion

Biofilm matrices are complex gel-like systems where biopolymers setup a network embedding cells and favouring proximity of interacting micro- and macro-molecular species. Among macromolecules, polysaccharides are considered to be the most relevant components to matrices architecture. This is due to their strong ability to interact within each other and with different macromolecules including proteins and extracellular DNA, to form the BF scaffold. Furthermore, polysaccharides may also exhibit specific biological properties which contribute to the protection offered to bacteria by biofilm matrices.

In this study, we analysed the influence of sub-inhibitory concentrations of Bac7(1–35) and BMAP-27 antimicrobial peptides on the biofilm forming capacity of two clinical isolates of *K. pneumoniae* and the protection exhibited by the matrices against these AMPs. In addition, the composition of exopolysaccharides present in the matrices was defined and the effect of their interaction with antimicrobial peptides was related with the protective effect.

The two *K. pneumoniae* isolates used for this investigation were chosen after screening over 30 strains exhibiting high variability in BF production. In particular, KpTs101 and KpTs113, although good BF producers, showed very different BF-adhesion properties: KpTs101 produced a biofilm strongly adhering to supports, whereas KpTs113 produced flocs. Therefore, all experiments that required adhesion to a surface were conducted only on KpTs101.

The adhesion properties of *K. pneumoniae* have been related to both type 3 fimbrial proteins and lipopolysaccharides [35]. Therefore, it can be speculated that the two strains investigated might exhibit differences in these molecular components. However, their role might be developed in synergy with matrix polysaccharides. In this study we demonstrated that the EPOL constituting the matrix are chemically very different. EPOL Kp101 is neutral and composed only of Gal residues. Interestingly, sugar rings are present both in the furanose and pyranose structures, indicating possible peculiar conformational features which might influence the overall polymer chain rigidity. On the contrary, EPOL Kp113 is a branched polysaccharide composed of mannose and glucose residues together with glucuronic acid. Thus, the negative charge exhibited by the polysaccharide might contribute to the scarce BF-matrix adhesion properties.

Sub-inhibitory concentrations of Bac7(1–35) and BMAP-27 affected biofilm production in a different manner, probably due to their different mechanism of action. Bac7(1–35) caused a decrease of biofilm production, even at much lower concentrations than MIC values (Figure 5A). This AMP is internalised and inhibits bacterial metabolism. Therefore, it can be speculated that bacteria, whose growth is partially inhibited (Figure 2A), are not able to release great amounts of exoproducts thus diminishing biofilm production. On the contrary, the effect of BMAP-27 treatment was more complex (Figure 5B) and dependent on the concentration used. At low concentration it inhibited biofilm production, but approaching the MIC value it induced an opposite effect, determining a higher amount of exoproducts. However, it is worth noting that results are expressed as biofilm index, i.e., the amount of biofilm normalised for bacterial growth. The results obtained suggest that at concentration very close to the MIC cells that remain able to grow must face a very hostile environment (indicated by the high variability detected among independent experiments) and synthesise larger amount of exoproducts than usual, probably in the attempt to protect themselves.

The investigation of the susceptibility of the two *K. pneumoniae* strains to antimicrobial peptides was preceded by the assessment of their mechanisms of action. Bac7(1–35) exerts its action by internalization and in fact flow cytometry assays (Figure 3) showed an effective internalization for both strains. BMAP-27 acts as bacterial membrane disruptor and flow cytometry experiments indicated that only at high AMP doses were both strains subjected to similar membranolytic effects (Figure 4). In the 0.25–0.50 µM range for BMAP-27, KpTs101 was subjected to membrane lysis to a greater extent than KpTs113. The susceptibility assays carried out with KpTs101 and KpTs113 in the presence of Bac7(1–35) and BMAP-27 proved the relevant protecting effect of biofilm, which enhanced resistance of embedded cells compared to the planktonic ones by about 30 times towards Bac7(1–35) and even more towards BMAP-27 (Table 1).

Surprisingly, investigating the effect of high concentrations of AMPs on the architecture of the biofilm gave opposite results (Figure 7), showing an intense crumbling effect by BMAP-27 compared to a moderate collapsing effect of Bac7(1–35). However, these experiments were conducted in saline solution where cells, blocked in the stationary phase, undergo the AMP’s action without opposing resistance. Compared to BIC evaluation, this condition weakens the effect of Bac7(1–35), which is almost inactive on non-replicating cells and only at high concentrations does it exert a moderate bacteriolytic effect which causes a sort of “precipitation” of dead cell to the deep layers of the biofilm. On the contrary, when mature biofilm under low-nutrient conditions was exposed to very high concentrations of BMAP-27, an evident disruption of the biomass was detected.

## 5. Conclusions

It is worth stressing that susceptibility assays carried out in the presence of polysaccharides extracted from BF of the two strains resulted in MIC values comparable to the BIC values obtained in the presence of native biofilms (Table 2, columns Ps). This protective effect shown by the extracted BF polysaccharides towards AMPs strongly suggested a direct interaction of the two molecular species. The interaction could be easily demonstrated for BMAP-27, via circular dichroism experiments carried out in phosphate buffer solutions, as it underwent a conformational transition from random coil to helical in the presence of both EPOL Kp101 and EPOL Kp113. As previously stated, the composition of the two exopolysaccharides is very different. The presence of the negatively charged glucuronic acid in the EPOL Kp113 could explain its helix-promoting activity in terms of electrostatic interactions with the cationic peptide. In fact, the formation of the amphipathic helix positions all cationic residues on the same side of the helix cylinder, thus increasing the efficiency of electrostatic interactions. The formation of secondary structure upon interaction with increasing concentrations of EPOL Kp113 nicely explains the decreased susceptibility of polysaccharide embedded cells with respect to planktonic ones, as it favours peptide self-aggregation and impedes proper contact with the bacterial surfaces [36]. Quite surprisingly, however, the neutral EPOL Kp101, exhibited an even better capacity to induce helix formation, indicating that interactions other than the electrostatic ones (e.g., H-bonding, hydrophobic and van der Waals interactions) are involved in the complexation with BMAP-27. In conclusion, both polysaccharides which contribute to the biofilm matrix composition exhibited a significant sequestering activity towards antimicrobial peptides with slightly different chemical mechanisms.

## Figures and Tables

**Table 1:** Biofilm production and susceptibility to antimicrobial peptides (AMPs) of *K. pneumoniae* strains.

BF ^1^ Production	No. of Strains	Representative Isolate	BF Index	Bac7(1–35)		BMAP-27	
				MIC	BIC	MIC	BIC
Excellent	2	KpTs101	145	2	64	2	>64
		KpMn7	101	4	32	4	64
Good	15	ATCC 700603	76	4	32	4	64
		KpTs107	67	4	16	4	>64
Scarce	13	KpTs12	13	4	8	4	16
		KpTs109	13	4	32	4	32
Flocs	1	KpTs113	-	2	64	2	>64

^1^ The ability to form biofilm of 30 clinical isolates and of ATCC 700603 strain was evaluated by the crystal violet method and categorized into three groups on the basis of the resulting biofilm index (BI). Excellent: BI ≥ 95th percentile; Good: 50th percentile ≤ BI < 95th percentile; Scarce: BI < 50th percentile. Flocs: see description of strain KpTs113 in the text. Susceptibility of planktonic and sessile cells of two strains representative of each group was evaluated by means of minimum inhibitory concentration (MIC) and biofilm inhibitory concentration (BIC), respectively. Inhibitory concentrations are expressed as µM.

**Table 2:** Antimicrobial activity of Bac7(1–35) and BMAP-27 towards KpTs101 and KpTs113 *K. pneumoniae* isolates. Activities are given as MIC for planktonic cells, as BIC for biofilms, and as MIC in the presence of matrix polysaccharides (Ps).

Strain	Bac7(1–35)			BMAP-27		
	MIC ^1^	BIC ^1^	Ps ^2^	MIC ^1^	BIC ^1^	Ps ^2^
KpTs101	2	64	32	2	>64	64
KpTs113	2	64	32	2	>64	64

^1^ MIC and BIC are expressed in µM; ^2^ Ps, MIC in the presence of 1 mg/mL of polysaccharide purified from the matrix.
